# MicroRNA-Mediated Regulation in Biological Systems with Oscillatory Behavior

**DOI:** 10.1155/2013/285063

**Published:** 2013-06-26

**Authors:** Zhiyong Zhang, Fengdan Xu, Zengrong Liu, Ruiqi Wang, Tieqiao Wen

**Affiliations:** ^1^Department of Mathematics, Shanghai University, Shanghai 200444, China; ^2^Institute of Systems Biology, Shanghai University, Shanghai 200444, China

## Abstract

As a class of small noncoding RNAs, microRNAs (miRNAs) regulate stability or translation of mRNA transcripts. Some reports bring new insights into possible roles of microRNAs in modulating cell cycle. In this paper, we focus on the mechanism and effectiveness of microRNA-mediated regulation in the cell cycle. We first describe two specific regulatory circuits that incorporate base-pairing microRNAs and show their fine-tuning roles in the modulation of periodic behavior. Furthermore, we analyze the effects of *miR369-3* on the modulation of the cell cycle, confirming that *miR369-3* plays a role in shortening the period of the cell cycle. These results are consistent with experimental observations.

## 1. Introduction

MicroRNAs are single-stranded non-coding RNA molecules containing 21~23-nucleotides. More and more works imply that microRNAs are involved in a series of important life processes, including early development, cell proliferation, differentiation, and apoptosis [1–11]. MicroRNAs act by base pairing with their target mRNAs and induce either translational repression or mRNA degradation through an RNA-induced silencing complex. Most microRNAs negatively regulate expression of their target genes. Since microRNA is a type of small molecules and needs not to be translated into proteins, it has an energy-saving advantage for the cell cycle regulation when compared to the regulation by proteins. In addition, its faster synthesis rate has more advantage in response to the changes in environment. These advantages mean that microRNA may play crucial roles in gene regulation.

A series of recent experiments show that microRNAs may play crucial roles in modulating periodic behaviors of biological systems, such as cell cycle and circadian rhythm [12–19]. Other reports indicate that microRNAs fine-tune oscillations of *p53* in the process of tumor suppression [20–29]. However, all these findings have still been confined to experimental stage. The operating mechanism and potential implication of microRNA-mediated regulation in the modulation of periodic behavior are less clear and need to be further investigated.

In this study, we aim to explore the control mechanism and kinetic characteristics of microRNA-mediated regulation in the modulation of cell cycle. First, we model two specific network motifs, which have different periodic behaviors in the absence of microRNA, that is, oscillation generated by a Hopf bifurcation and relaxation oscillation. Furthermore, microRNA is incorporated into these two motifs, respectively. The dynamical analysis confirms that microRNA can regulate these two types of oscillations by shortening their periods. Then we study the microRNA regulation of a periodic phenomenon in biological system, that is, cell cycle. The results account for the roles of microRNA in the modulation of cell cycle observed in recent experiments.

## 2. MicroRNA Regulation of Two Motifs and Cell Cycle

### 2.1. Analysis of Motif I

**Figure 1:**
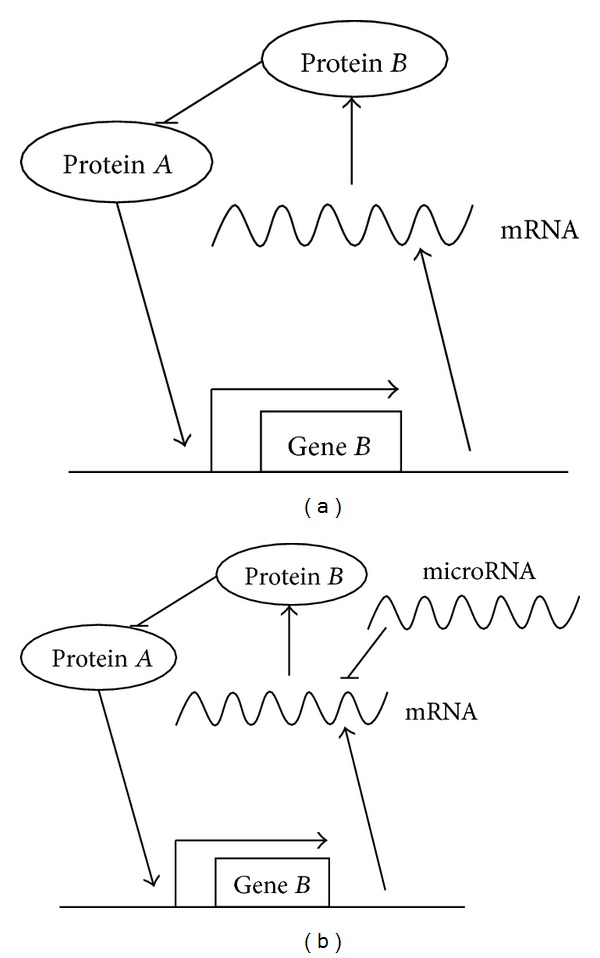
Schematic description of the first motif without and with microRNA regulation. (a) Without microRNA regulation; (b) with microRNA regulation.

 The first motif without and with the regulation of microRNA is shown in Figure 1. In this motif, protein *A* activates the transcription of gene *B*, which in turn inhibits the transcription of gene *A*, as shown in Figure 1(a).

With these assumptions, when the microRNA is not incorporated, we get a set of differential equations on the concentrations of the substrates, which describe the behaviors of the mRNA of gene *B*, protein *B*, and protein *A*, as follows:

(1) $$\begin{array}{c} \frac{d\left[m\right]}{dt}=a_{1}f\left(\left[m\right]\right)-d_{m}\left[m\right],\\ \frac{d\left[B\right]}{dt}=a_{2}\left[m\right]-d_{B}\left[B\right],\\ \frac{d\left[A\right]}{dt}=a_{3}g\left(\left[B\right]\right)-d_{A}\left[A\right], \end{array}$$

where [·] means the concentration of the substrate, and *f*([*A*]) = [*A*]^*n*_1_^/([*A*]^*n*_1_^ + *K*_*A*_^*n*_1_^), *g*([*B*]) = *K*_*B*_^*n*_2_^/([*B*]^*n*_2_^ + *K*_*B*_^*n*_2_^), where *n*_1_, *n*_2_ are hill coefficients.

When the microRNA regulation is incorporated into (1) (see Figure 1(b)), it becomes

(2) $$\begin{array}{c} \frac{d\left[m\right]}{dt}=a_{1}f\left(\left[m\right]\right)-d_{m}\left[m\right]-d\left[m\right]\left[s\right],\\ \frac{d\left[B\right]}{dt}=a_{2}\left[m\right]-d_{B}\left[B\right],\\ \frac{d\left[A\right]}{dt}=a_{3}g\left(\left[B\right]\right)-d_{A}\left[A\right],\\ \frac{d\left[s\right]}{dt}=\lambda -d_{s}\left[s\right]-d\left[m\right]\left[s\right], \end{array}$$

where *λ* is the synthesis rate of microRNA, *d*_*s*_ is the degradation rate of microRNA, and *d* is the associate rate of two RNAs.

Using the dimensionless variables scaled by *τ* = *a*_1_ *t*, $\hat{B}=d[B]/K_{B}$ , $\hat{A}=d[A]/K_{A}$ , ${\hat{a}}_{2}=a_{2}/a_{1}K_{B}$ , ${\hat{a}}_{3}=a_{3}/a_{1}K_{A}$ , ${\hat{d}}_{m}=d_{m}/a_{1}$ , ${\hat{d}}_{B}=d_{B}/a1$ , ${\hat{d}}_{A}=d_{A}/a1$ , $\hat{f}(\hat{A})={\hat{A}}^{n_{1}}/\left({\hat{A}}^{n_{1}}+1\right)$ , $\hat{g}(\hat{B})=1/\left({\hat{B}}^{n_{2}}+1\right)$ , and $\hat{\lambda }=\lambda /a_{1}$ , ${\hat{d}}_{s}=d_{s}/a_{1}$ , $\hat{d}=d/a_{1}$ , we obtain two sets of equations as follows:

(3) $$\begin{array}{c} \frac{d\left[m\right]}{d\tau }=\hat{f}\left(\hat{A}\right)-{\hat{d}}_{m}\left[m\right],\\ \frac{d\hat{B}}{d\tau }={\hat{a}}_{2}\left[m\right]-{\hat{d}}_{B}\hat{B},\\ \frac{d\hat{A}}{d\tau }={\hat{a}}_{3}\hat{g}\left(\hat{B}\right)-{\hat{d}}_{A}\hat{A}, \end{array}$$

(4) $$\begin{array}{c} \frac{d\left[m\right]}{d\tau }=\hat{f}\left(\hat{A}\right)-{\hat{d}}_{m}\left[m\right]-\hat{d}\left[m\right]\left[s\right],\\ \frac{d\hat{B}}{d\tau }={\hat{a}}_{2}\left[m\right]-{\hat{d}}_{B}\hat{B},\\ \frac{d\hat{A}}{d\tau }={\hat{a}}_{3}\hat{g}\left(\hat{B}\right)-{\hat{d}}_{A}\hat{A},\\ \frac{d\left[s\right]}{d\tau }=\hat{\lambda }-{\hat{d}}_{s}\left[s\right]-\hat{d}\left[m\right]\left[s\right]. \end{array}$$

We can calculate the equilibriums by setting the right-hand sides of the equations equal to zero. Let the equilibrium be (m*, B*, A*) for (3). Linearizing (3) around the equilibrium results in the Jacobian matrix as follows:

(5) $$\begin{array}{c} J_{1}=\left(\begin{array}{ccc} -{\hat{d}}_{m} & 0 & {\hat{f}}^{\prime }\left(A^{*}\right)\\ {\hat{a}}_{2} & -{\hat{d}}_{B} & 0\\ 0 & {\hat{a}}_{3}{\hat{g}}^{\prime }\left(B^{*}\right) & -{\hat{d}}_{A} \end{array}\right). \end{array}$$

By simple calculation, its characteristic polynomial is derived as follows:

(6) x3+(d^m+d^B+d^A)x2+(d^md^B+d^md^A+d^Bd^A)x +d^md^Bd^A−a^2a^3f^′(A∗)g^′(B∗)=0.

Using Routh-Hurwitz criteria, we know that when the following condition is satisfied:

(7) (d^m+d^B+d^A)(d^md^B+d^md^A+d^Bd^A) =d^md^Bd^A−a^2a^3f^′(A∗)g^′(B∗),

a Hopf bifurcation occurs (and the right-hand side should be also larger than 0). In other words, when the left-hand side becomes less than the right-hand side in the above equation, oscillations will occur in (3).

Let the equilibrium be (*m*°, *B*°, *A*°, *s*°) for (4). Similarly, linearizing (4) around the equilibrium results in the Jacobian matrix as follows:

(8) $$\begin{array}{c} J_{2}=\left(\begin{array}{cccc} -{\hat{d}}_{m}-\hat{d}s^{\circ } & 0 & {\hat{f}}^{\prime }\left(A^{\circ }\right) & -\hat{d}m^{\circ }\\ {\hat{a}}_{2} & -{\hat{d}}_{B} & 0 & 0\\ 0 & {\hat{a}}_{3}{\hat{g}}^{\prime }\left(B^{\circ }\right) & -{\hat{d}}_{A} & 0\\ -\hat{d}s^{\circ } & 0 & 0 & -{\hat{d}}_{s}-\hat{d}m^{\circ } \end{array}\right). \end{array}$$

By calculation, its characteristic polynomial is derived as follows:

(9) $$\begin{array}{c} x^{4}+\alpha_{1}x^{3}+\alpha_{2}x^{2}+\alpha_{3}x+\alpha_{4}=0, \end{array}$$

where $\alpha_{1}={\hat{d}}_{m}+{\hat{d}}_{B}+{\hat{d}}_{A}+{\hat{d}}_{s}+\hat{d}s^{\circ }+\hat{d}m^{\circ }$ , $\alpha_{2}=\hat{d}s^{\circ }{\hat{d}}_{B}+\hat{d}s^{\circ }{\hat{d}}_{A}+{\hat{d}}^{2}s^{\circ }m^{\circ }+{\hat{d}}_{B}\hat{d}m^{\circ }+\hat{d}s^{\circ }{\hat{d}}_{s}+{\hat{d}}_{B}{\hat{d}}_{s}+{\hat{d}}_{A}{\hat{d}}_{s}+{\hat{d}}_{m}\hat{d}m^{\circ }-{\hat{d}}^{2}{m^{\circ }}^{2}+{\hat{d}}_{m}{\hat{d}}_{A}+{\hat{d}}_{B}{\hat{d}}_{A}+{\hat{d}}_{m}{\hat{d}}_{s}+{\hat{d}}_{m}{\hat{d}}_{B}+{\hat{d}}_{A}\hat{d}m^{\circ }$ , and so on. Again, according to the Routh-Hurwitz criteria, we can obtain the condition of Hopf bifurcation as follows:

(10) $$\begin{array}{c} \Delta_{3}=\left|\begin{array}{ccc} \alpha_{1} & \alpha_{3} & 0\\ 1 & \alpha_{2} & \alpha_{4}\\ 0 & \alpha_{1} & \alpha_{3} \end{array}\right|=0, \end{array}$$

that is, when the determinant becomes less than zero (and *α*_1_ > 0, *α*_3_ > 0, *α*_4_ > 0), oscillations will occur in (4).

To investigate the dynamical properties of microRNA regulation in gene expression, we will next examine and compare the two models by computational analysis. To determine fundamental differences in these two models without and with microRNA regulation, it would be of interest to choose similar parameter values. We first choose values of parameters in (3), under which oscillations may occur. Then, under these fixed parameter values, other values of parameter in (4) are chosen so as to produce oscillations.

**Figure 2:**
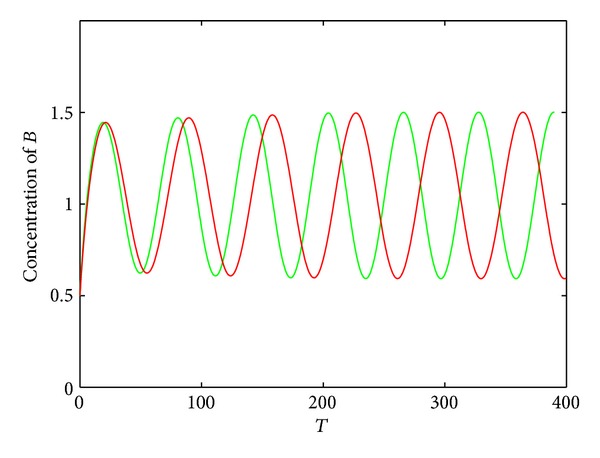
The modulation of oscillations by microRNA in motif I. The green and red lines show the concentration of protein *B* with and without microRNA regulation, respectively.

In Figure 2, the following parameter values in (3) are used: *n*_1_ = 2, *n*_2_ = 2, ${\hat{a}}_{2}=1.4$ , ${\hat{a}}_{3}=3.4$ , ${\hat{d}}_{m}=0.1$ , ${\hat{d}}_{B}=0.1$ , and ${\hat{d}}_{A}=0.15$ . Other parameter values in (4) are $\hat{\lambda }=2.0$ , ${\hat{d}}_{s}=0.2$ , and $\hat{d}=0.8$ . It can be seen that after the incorporation of the microRNA regulation, the changes of the wave form and amplitude of the oscillation are slight. In contrast, the period is shortened evidently, which means that the fine-tuning of microRNA regulation in the oscillation is period shortening.

### 2.2. Analysis of Motif II

**Figure 3:**
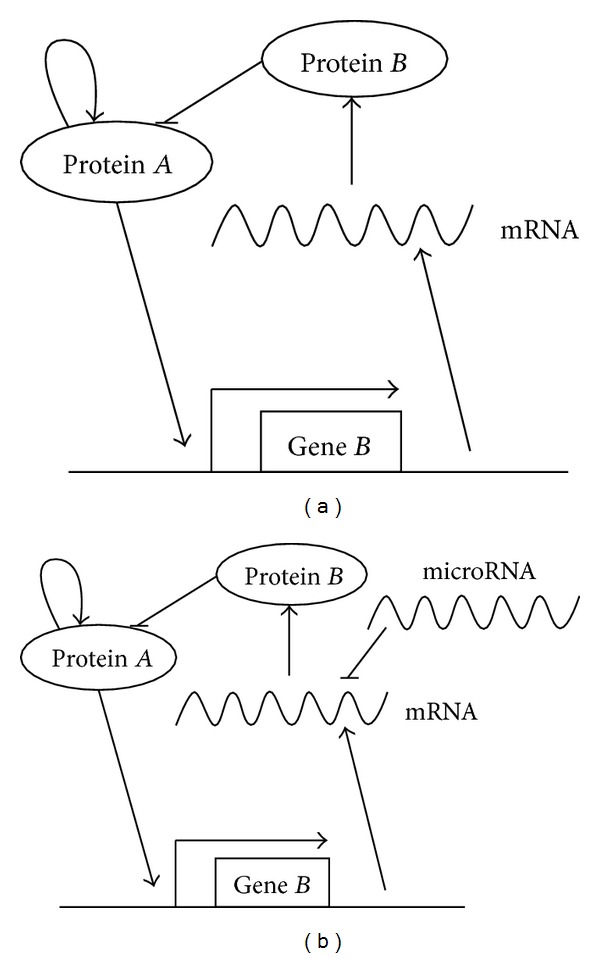
Schematic description of the second motif without and with microRNA regulation. (a) Without microRNA regulation; (b) with microRNA regulation.

The second motif is similar to the first one except the positive autoregulation of protein *A*, as shown in Figure 3.

When the microRNA is not incorporated, it has been discussed in [30]. It was shown that relaxation oscillation could occur due to the existence of different time scales. Using their model, the system without microRNA regulation can be expressed as follows:

(11) $$\begin{array}{c} \frac{dA}{dt}=\Delta \left(a_{1}+\frac{1+\rho A^{2}}{1+\rho A^{2}+\sigma B^{2}}-A\right),\\ \frac{dB}{dt}=\Delta a_{2}\frac{1+\rho A^{2}}{1+A^{2}}-B, \end{array}$$

where Δ is the ratio of degradation rates between the two proteins *A* and *B*, Δ ≫ 1, which means degradation rate of *A* is much faster than that of *B*. *α*_2_ = *ϵα*_1_ with 0 < *ϵ* ≪ 1 means that the synthesis rate of protein *B* is much slower than that of *A*. Similar to the first motif, after the incorporation of miRNA regulation, the system can be rewritten as follows:

(12) $$\begin{array}{c} \frac{dA}{dt}=\Delta \left(a_{1}+\frac{1+\rho A^{2}}{1+\rho A^{2}+\sigma B^{2}}-A\right),\\ \frac{dB}{dt}=\Delta a_{2}\frac{1+\rho A^{2}}{1+A^{2}}-B-dBs,\\ \frac{ds}{dt}=\lambda -d_{s}s-dBs, \end{array}$$

where the first equation means that microRNA *s* and protein *B* codegrade nonlinearly at a rate *d* besides their respective linear degradation. Similarly, the parameter values in (11) are chosen first so as to produce relaxation oscillations. Then other parameters in (12) are chosen. More exactly, parameter values are *a*_1_ = 1.58, *a*_2_ = 0.05*a*_1_, *ρ* = 50, *σ* = 1, Δ = 11, *λ* = 5, *d*_*s*_ = 0.1, and *d* = 0.1.

**Figure 4:**
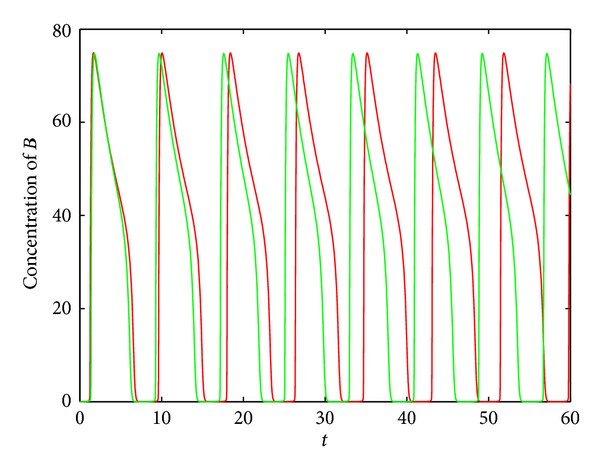
The modulation of relaxation oscillation by microRNA in motif II. The green and red lines show the concentration of protein *B* with and without microRNA regulation, respectively.

 When the microRNA is not incorporated, relaxation oscillation occurs, as shown by the red line in Figure 4. Similar to the first motif, after the incorporation of the microRNA, the changes of the wave form and amplitude of the relaxation oscillation are slight. In contrast, the period is shortened significantly too, meaning that the main modulation of microRNA regulation in relaxation oscillation is also period shortening.

For both of the motifs, we get similar results; that is, the main modulation of microRNA regulation in periodic behavior is period shortening. In contrast, the types of oscillations, the wave forms, and amplitude of oscillations do not change significantly, thereby fine-tuning periodic behavior of biological systems.

### 2.3. Cell Cycle Regulation

Next, we study the roles of microRNA in modulation of cell cycle by incorporating a microRNA, that is, *miR369-3*, into a detailed model.

Recently, experimental report by Vasudevan et al. revealed that microRNA can up-regulate gene expression in G0/G1 arrest during mammalian cell cycle [31]. In fact, they carried out a series of experiments by connecting a reporter mRNA to study *miR369-3* expression in different conditions. These results show that *miR369-3* promotes translation in G0/G1 arrest and suppresses translation in proliferating cells. It is equivalent to shortening the cell cycle process from the perspective of the entire cell cycle.

Many excellent theoretical models have been proposed to study the cell cycle [32–45]. The cell cycle consists of four phases: G1 phase, S phase, G2 phase, and M phase. Activation of the next stage is dependent on the normal completion of previous one. In [32], Novák and Tyson model the cell cycle as a two-steady-state process in which the two steady states are corresponding to G1 stage and S/G2/M stage, respectively. And the periodic switching between these two stable states is a result of the antagonism between CycB/Cdk1 and Cdh1/APC, which creates a G1 state with active Cdh1 and low CycB activity and an S/G2/M state with high CycB level and Cdh1 turned off.

Here we develop a computational model based on Tyson's cell cycle model [32] to investigate the posttranscriptional role of microRNA in the modulation of the mammalian cell cycle. By using computational target prediction algorithm miRBase [46], we screen out all the potential target genes of *miR369-3*; all of them are not directly in the pathway given in Tyson's model. However, among these potential target genes, *Lox* is found to inactivate CycD, an important gene in cell cycle during the period G0/G1 [47]. Therefore, by introducing the new pathway *miR369-3-Lox-CycD* into the Tyson's model, miR369-3 is incorporated into the cell cycle model.

Now, let us study the regulatory effects of microRNA in the modified model with kinetic analysis. The dynamical equations are different from Tyson's model in two points: two new equations for Lox and *miR369-3* and one additional term in equation for CycD to show the inactivation of CycD by Lox. Refer to following equations:


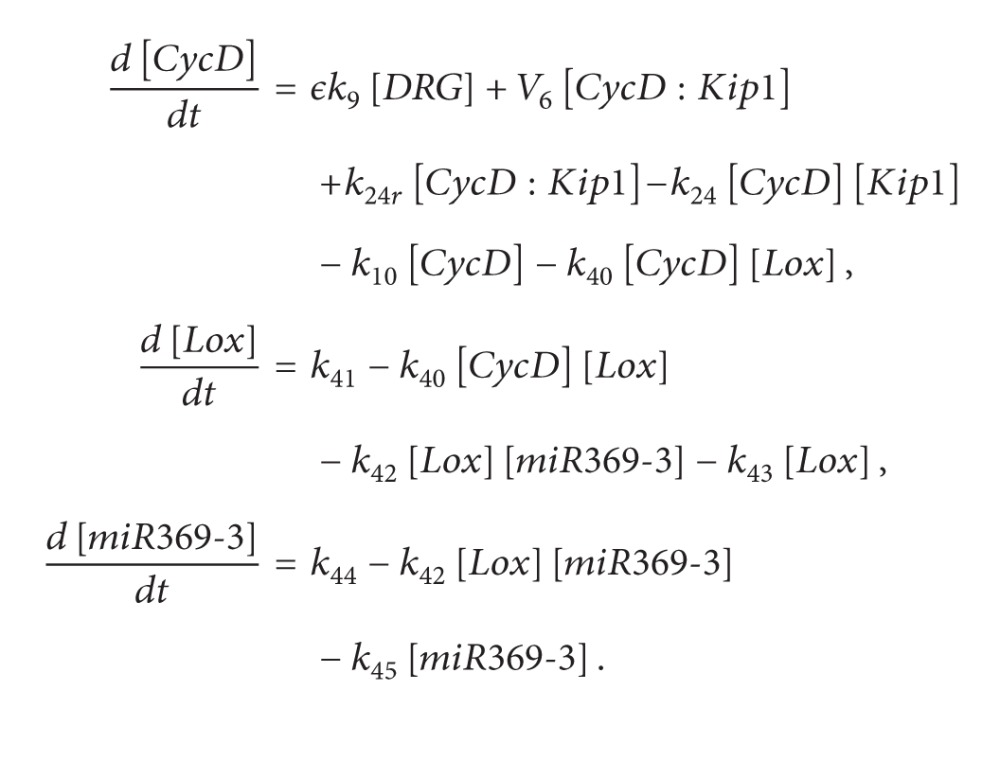

(13)



The new parameters *k*_44_, *k*_45_, *k*_42_, *k*_41_, *k*_43_, and *k*_40_ are the production rate of *miR369-3*, linear degradation rate of *miR369-3*-association rate of *miR369-3* with *Lox*, production rate of *Lox*, linear degradation rate of *Lox*, and inactivation rate of *CycD* by *Lox*, respectively.

Next, numerical simulations are conducted. In the simulations, all parameter values are the same as those used in Tyson's model except the newly introduced ones. We run the simulation with a wide range of the new parameters, and similar results are derived. Here we show the results with the following set of parameters. Before the incorporation of the microRNA, the parameter values are taken as *k*_40_ = 50, *k*_41_ = 200, *k*_42_ = 0, *k*_43_ = 5, *k*_44_ = 0, and *k*_45_ = 0. After incorporating it, they are *k*_40_ = 50, *k*_41_ = 200, *k*_42_ = 80, *k*_43_ = 5, *k*_44_ = 200, and *k*_45_ = 5.

**Figure 5:**
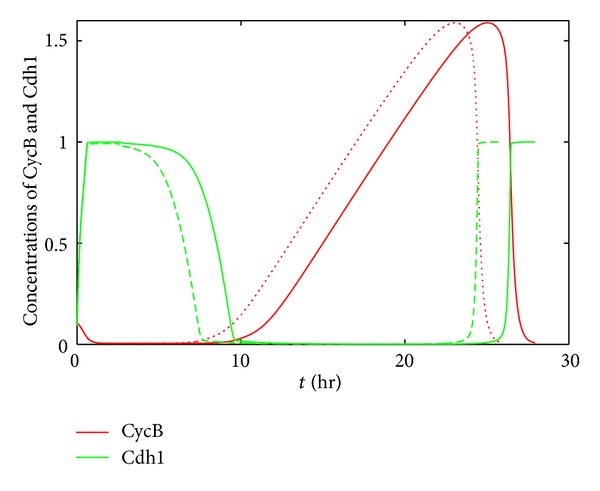
The modulation of cell cycle by *miR369-3*. The dashed and solid lines show the concentration of CycB and Cdh1 with and without microRNA regulation, respectively.

A simulation with and without microRNA regulation is presented in Figure 5, where concentrations of CycB and Cdh1 are shown. It can be seen that the incorporation of miR369-3 shortens the period of CycB with low expression representing the G1 phase and cells enter M phase earlier, which is in agreement with the experimental observations. We infer from the above results that miR369-3 exerts its regulation on cell cycle through the cell cycle period modulation. This founding is also similar to the results for the two motifs studied above.

**Figure 6:**
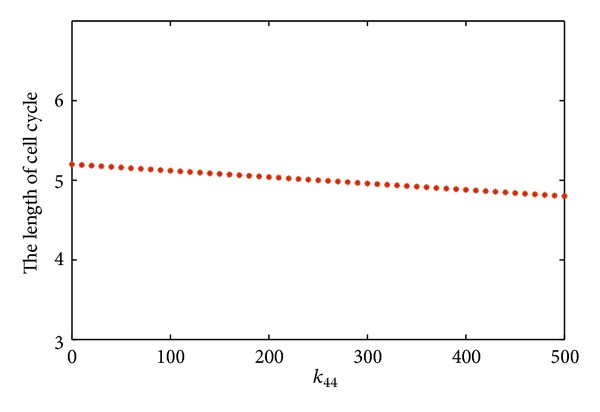
The effect of production rate of miR369-3 on the length of the cell cycle.

The linear nature of the dependence of period on progressively varied *k*_44_ is shown in Figure 6, which further reflects the roles played by the microRNA-mediated regulation. The period of oscillations decreases slightly but almost linearly with *k*_44_, meaning the fine-tuning of cell cycle by the miR369-3.

## 3. Discussion

MicroRNA-mediated regulation has gained recent attention. With the accumulation of our knowledge about microRNA, more and more regulatory mechanisms will be revealed.

Recent experimental works have implicated that microRNAs may play a fine-tuning role in cell cycle regulation. However, most of them are based on experimental works, and the regulatory mechanisms are less clear theoretically and need to be further investigated.

Beginning with two motifs, we find that microRNAs do not significantly change the type of oscillations, including their wave forms and amplitudes, while the period changes significantly. It seems that such a fine-tuning is general for both motifs. With those results for simplified models, we further incorporate a microRNA, *miR369-3*, into a classical cell cycle model and study the microRNA regulatory effects and mechanisms for cell cycle. We found that the *miR369-3* regulates cell cycle by slightly shortening the cell cycle period, thus accelerating the cell cycle, which is in agreement with experimental observations. With the accumulatation of experimental results related to microRNA regulation in cell cycle, we will further incorporate more microRNAs into cell cycle and investigate the overall regulatory effects of microRNAs regulation in the future, which may give deep insights into microRNA regulatory mechanisms in cell cycle.

Finally, it is worth noting that microRNA-mediated regulation has gained recent attention, and computational studies have revealed various regulatory properties unique to microRNAs. These findings will be helpful for our understating the operating mechanisms and biological implications of microRNA-mediated regulation. They also have great potential for biotechnological, therapeutic applications, and synthetic biology.
